# Identification of Hub Genes and Analysis of their Regulatory miRNAs in Patients with Thymoma Associated Myasthenia Gravis Based on TCGA Database

**DOI:** 10.2174/0122115366299210240823062457

**Published:** 2024-08-26

**Authors:** Wei Zhou, Jia Hu, Jun Nie

**Affiliations:** 1 Department of Cardiothoracic Surgery, The First Affiliated Hospital of Wannan Medical College, Wuhu, China;; 2 Department of Neurology, The First Affiliated Hospital of Wannan Medical College, Wuhu, China

**Keywords:** Myasthenia gravis, thymoma, immune cell infiltration, KWs, KEGG, microrna

## Abstract

**Background:**

Myasthenia gravis is an autoimmune disease, and 30% of patients with thymoma often have myasthenia gravis. Patients with thymoma-associated MG (TAMG) have many different clinical presentations compared to non-MG thymoma (NMG), yet their gene expression differences remain unclear.

**Objective:**

In this study, we analyzed the Differentially Expressed Genes (DEGs) and analyzed their regulatory microRNAs (miRNAs) in TAMG, which will further clarify the possible pathogenesis of TAMG.

**Methods:**

DEGs were calculated using the RNA-sequencing data of TAMG and NMG downloaded from The Cancer Genome Atlas (TCGA) database. R software was then used to analyze the gene ontology (GO) and Kyoto Encyclopedia of Genes and Genomes (KEGG) pathways of DEGs, while STRING was applied to build the protein-protein interaction (PPI) network and Cytoscape to identify and visualize the hub genes. Immune infiltration significances of hub genes were also explored by using the TIMER database and TCGA database. Upstream microRNAs (miRNAs) of the hub genes were predicted by online software.

**Results:**

We comparatively analyzed the gene expression differences between TAMG and NMG groups. A total of 977 DEGs were identified between the two groups (|log fold change (FC)| >2, adjusted P value <0.050), with 555 down-regulated genes and 422 up-regulated genes. Five top hub genes (CTNNB1, EGFR, SOX2, ERBB2, and EGF) were recognized in the PPI network. Analysis based on the TIMER and TCGA databases suggested that 5 hub genes were correlated with multiple immune cell infiltrations and immune checkpoint-related markers, such as PDCD1, CTLA-4, and CD274, in TAMG patients. Lastly, 5 miRNAs were identified to have the potential function of regulating the hub gene expression.

**Conclusion:**

Our study identified 5 hub genes (CTNNB1, EGFR, SOX2, ERBB2, and EGF) and their 5 regulatory miRNAs in TAMG, and the hub genes were correlated with multiple immune cell infiltrations and immune checkpoint-related markers. Our findings could help partially clarify the pathophysiology of TAMG, which could be new potential targets for subsequent clinical immunotherapy.

## 1 INTRODUCTION

Thymoma is a type of thymic epithelial tumor that originates from thymic epithelial cells. With about 1.5 instances per million persons in the US, its prevalence is comparatively low [1]. Thymoma is the most common pathological type of anterior mediastinal tumor, accounting for about half of anterior mediastinal tumors [2, 3]. Previous studies showed that 30% of patients with thymoma often have Myasthenia Gravis (MG), which is one of the paraneoplastic autoimmune diseases [4, 5]. According to WHO histological classification, patients with type B thymoma are more likely to have a combination of MG, especially type B2 [6].

Myasthenia gravis is an autoimmune disease in which patients often feel weak and fatigued. The disease is characterized by the production of autoantibodies against AChR, Musk, and LRP4 in the patient's body [7]. The current treatment for thymoma-associated MG (TAMG) mainly consists of thymectomy, immunosuppression, glucocorticoids, and plasma exchange [8, 9]. In patients with TAMG, alleviating the symptoms of myasthenia gravis is of great clinical importance to improve the quality of life and survival time of patients [10]. Since myasthenia gravis is an autoimmune disease, it is important to understand the immune cell infiltration for its treatment. A recent study showed that patients with MG had fewer CTLA4-positive cells in the thymoma than non-MG thymoma (NMG) patients [11]. However, the immune cell infiltration of TAMG patients has not been fully elucidated currently.

In this study, we used bioinformatics methods to study the RNA-sequencing data of thymoma patients in the TCGA database. Differentially expressed genes (DEGs) were analyzed between TAMG and NMG patients, and 5 hub genes were then identified [12]. R software was adopted to explore the correlation between key genes and immune infiltrating cells by employing the TIMER database and TCGA database. Then, we constructed the hub genes-miRNAs interactions network. Our findings could help partially clarify the pathophysiology of TAMG, which could be new potential targets for subsequent clinical immunotherapy.

## 2 METHODS

### 2.1 TCGA Database Analysis

The Cancer Genome Atlas (TCGA)(https://www.cancer.gov/ccg/research/genome-sequencing/tcga), a landmark cancer genomics program, has molecularly characterized more than 20,000 primary cancer and matched normal samples across 33 cancer types. RNA sequencing data of thymoma patients with corresponding clinical information were downloaded from the Genomic Data Commons (https://portal.gdc.cancer.gov/). According to whether they suffered from myasthenia gravis, all the patients with thymoma (*n*=117) were divided into two groups: TAMG group (*n*=83) and NMG group (*n*=34) [11].

### 2.2 Analysis of Differentially Expressed Genes (DEGs)

We used R software (version 4.2.1), and variance analysis of the original counts matrix of mRNA was carried out by the DESeq2 package (version 1.36.0), following the standard process. Normalization of the original counts matrix was processed by the VST (Variance Stabilizing Transformations) method provided by the DESeq2 package (|log fold change (FC)| >2, adjusted P value <0.05) [12, 13]. Volcano plots were adopted to visualize DEGs by using the ggplot2 R package.

### 2.3 GO and KEGG Pathway Analyses

To explore the function and metabolic pathways of DEGs between the TAMG group and NMG group, Gene Ontology (GO) and Kyoto Encyclopedia of Genes and Genomes (KEGG) analyses were performed using the ClusterProfiler R package (version 4.4.4). Bubble charts were applied to visualize the top 5 enriched terms of cellular component (CC), molecular function (MF), biological process (BP), and KEGG pathways [14].

### 2.4 Protein-protein Interactions (PPI) Networks and Hub Genes

STRING (version 11.5) is an online database (https://cn.string-db.org/) of known and predicted protein-protein interactions. DEGs were inputted into the STRING database. Then, the interactions, including direct (physical) and indirect (functional) associations, were visualized online [15, 16]. The cytoHubba app of Cytoscape software (version 3.9.1) was used to find the hub genes in the PPI network. The top 5 genes ranked by degree were then calculated and visualized [17].

### 2.5 Immune Infiltration Analysis

TIMER is an online tool (https://cistrome.shinyapps.io/ti-mer/) to analyze immune cell infiltration in different types of cancer. The abundances of 6 types of immune cell infiltration (B cells, CD4^+^ T cells, CD8^+^ T cells, neutrophils, macrophages, and dendritic cells) in thymoma were quantified using the TIMER algorithm.

Immune cell infiltration was also explored using a single sample Gene Set Enrichment Analysis (ssGSEA). The mRNA-seq data of thymoma in the TCGA database was calculated using the GSVA R package. This approach allows the analysis of the correlation of 24 immune cells and hub genes, which is presented using the ggplot2 R package [18, 19].

### 2.6 Hub Genes-miRNAs Interactions Analysis

NetworkAnalyst, an online database, was used to construct the hub genes-miRNAs interactions network [20].

### 2.7 Statistical Analysis

All statistical analysis and plots in this study were calculated using R software (version 4.2.1) and GraphPad Prism 9.0. *P*<0.05 was considered statistically significant in all the tests.

## 3 RESULTS

### 3.1 Identification of DEGs

**Fig. (1):**
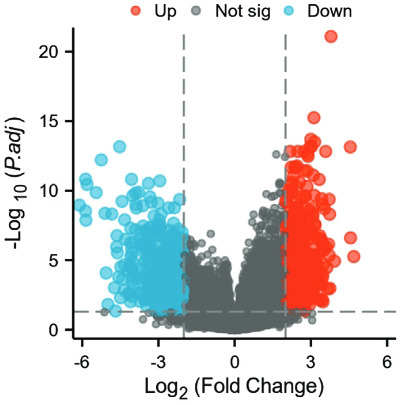
Volcano plot of DEGs between TAMG and NMG groups (|log FC| >2, adjusted P value <0.05). Red dots represent up-regulated genes, while blue dots represent down-regulated genes. TAMG, thymoma-associated myasthenia gravis; NMG, non-MG thymoma.

By R software, we comparatively analyzed DEGs between TAMG and NMG groups. Finally, 977 genes were proved to be significantly different between the two groups (|log fold change (FC)| >2, adjusted *P* value <0.05), with 555 down-regulated genes and 422 up-regulated genes. All the DEGs were shown by using a volcano plot (Fig. **1**).

### 3.2 GO and KEGG Analyses of DEGs

**Fig. (2):**
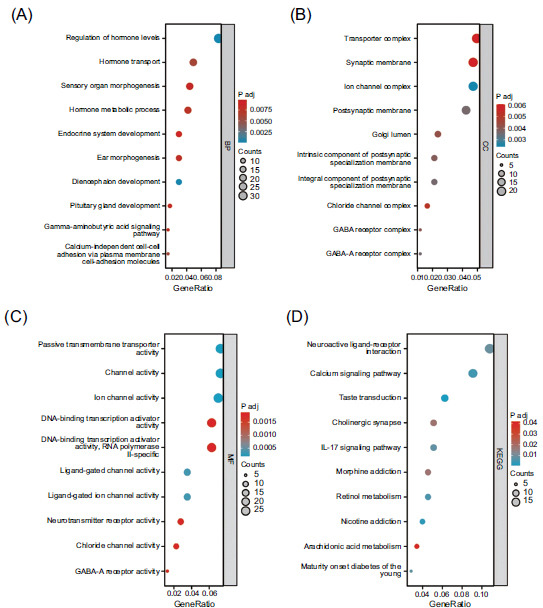
GO and KEGG analyses of DEGs. Go functional analysis of DEGs in three functional groups: biological process (**A**), cellular component (**B**), and molecular function (**C**). KEGG pathway enriched analysis of DEGs. (**D**) GO, Gene Ontology; KEGG, Kyoto Encyclopedia of Genes and Genomes. DEGs, Differentially Expressed Genes.

To explore the function of the DEGs, we performed GO and KEGG analyses. As shown in Fig. (**2**), the top 10 enriched entries were demonstrated. The mainly enriched items for biological process (BP) were regulation of hormone levels, hormone transport, sensory organ morphogenesis, hormone metabolic process, and endocrine system development (Fig. **2A**). Considering cellular component (CC), the mainly enriched items were transporter complex, synaptic membrane, ion channel complex, postsynaptic membrane, and Golgi lumen (Fig. **2B**). The molecular function (MF) of DEGs mainly included passive transmembrane transporter activity, channel activity, ion channel activity, DNA-binding transcription activator activity, and RNA polymerase II-specific (Fig. **2C**). Mainly enriched KEGG pathway items were neuroactive ligand-receptor interaction, calcium signaling pathway, taste transduction, cholinergic synapse, and IL-17 signaling pathway (Fig. **2D**).

### 3.3 PPI network and Hub Gene Identification

**Fig. (3):**
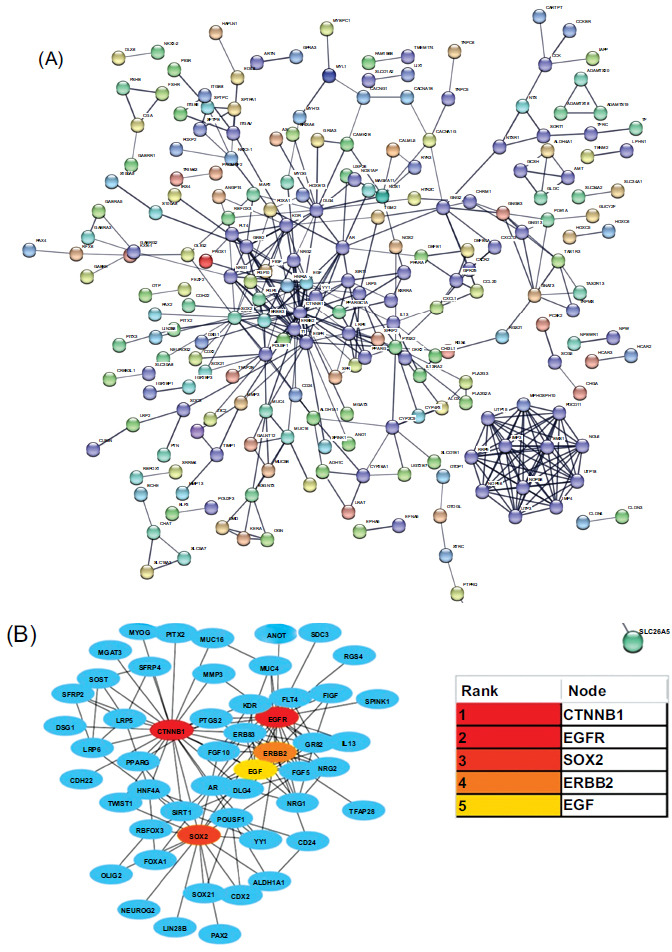
PPI network of DEGs and hub genes. (**A**) PPI network drawn by STRING database. (**B**) Five hub genes identified by the cytoHubba application based on Cytoscape software.

STRING database was used to analyze the protein-protein interaction of DEGs. As shown in Fig. (**3A**), 443 nodes and 806 edges were identified in the PPI network (Fig. **3A**). For the determination of the key role genes in the network, the cytoHubba application based on Cytoscape software was used. Degrees of each node were calculated and ordered from high to low, and then the top 5 hub genes were identified: CTNNB1, EGFR, SOX_2_, ERBB_2_, and EGF (Fig. **3B**).

### 3.4 The Correlation Between Hub Gene Expression and Immune Cell Infiltration

The TIMER database was used to explore the correlation between 5 hub genes and immune infiltration in TAMG patients. As shown in Fig. (**4**), CTNNB1 expression level was negatively correlated with CD8^+^ T cells infiltration level, while it was significantly positively correlated with the infiltration level of B cells, CD4^+^ T cells, macrophage, neutrophil, and dendritic cells. EGFR expression was negatively correlated with CD8^+^ T cells infiltration level, while it was significantly positively correlated with infiltration levels of B cells, CD4^+^ T cells, macrophage, neutrophil, and dendritic cells. SOX2 expression level showed a very weak positive correlation with CD4^+^ T cells and neutrophil cells. ERBB2 expression level was negatively correlated with CD8^+^ T cells infiltration level, while it was significantly positively correlated with infiltration levels of B cells. EGF expression level showed a very weak correlation with the infiltration levels of B cells, neutrophils, and dendritic cells.

We then performed appropriate analysis and verification using the ssGSEA method. The level of infiltration of 24 immune cell types in thymoma patients was calculated by analyzing RNA-seq data in the TCGA database. As shown in Fig. (**5**), CTNNB1 expression was strongly positively correlated with NK cells, macrophages, and Tgd cells but negatively correlated with T cells, TFH, pDC, Th17 cells, DC, and B cells. EGFR was strongly positively correlated with NK cells but negatively correlated with NK CD56dim cells, TFH, B cells, T cells, DC, Th2 cells, and Th17 cells. SOX_2_ was strongly positively correlated with NK cells but negatively correlated with CD8^+^ T cells, B cells, TFH, and Th17 cells. ERBB2 was strongly positively correlated with NK cells and macrophage cells but negatively correlated with Tcm, pDC, CD8^+^ T cells, TFH, T cells, Th17 cells, DC, and B cells. EGF was strongly positively correlated with NK cells, macrophages, and Tgd cells but negatively correlated with T cells, TFH, pDC, Th17 cells, DC, and B cells (supplementary material).

### 3.5 The relationship between hub genes and the immune checkpoint-related molecules

**Fig. (5):**
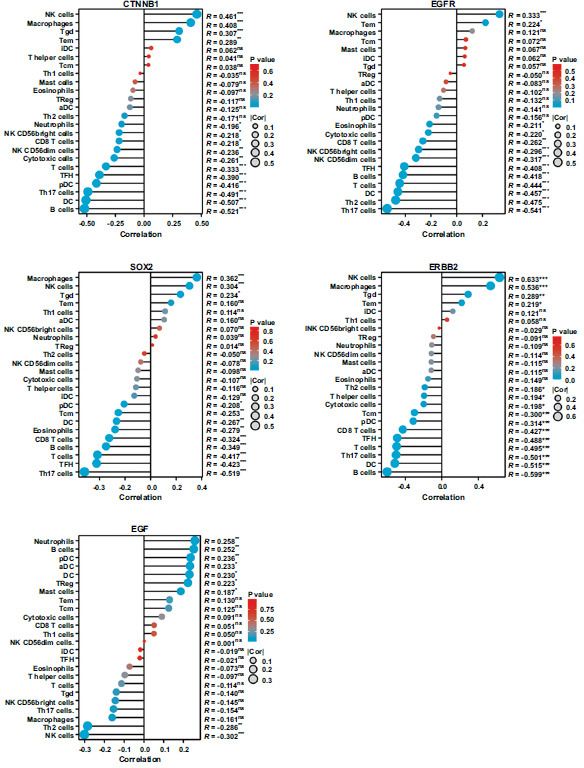
Correlation between the hub genes expression level and 24 immune cells. The size of the bubbles represents the value of Spearman. **Abbreviations:** NK cells, natural killer cells; DC, dendritic cell; pDC, plasmacytoid dendritic cell; iDC, immature DC; aDC, activated DC; Th, helper T cells; Treg, regulatory T cells; Tem, T effector memory; Tfh, T follicular helper; Tcm, T central memory; Tgd, T gamma delta.

**Fig. (6):**
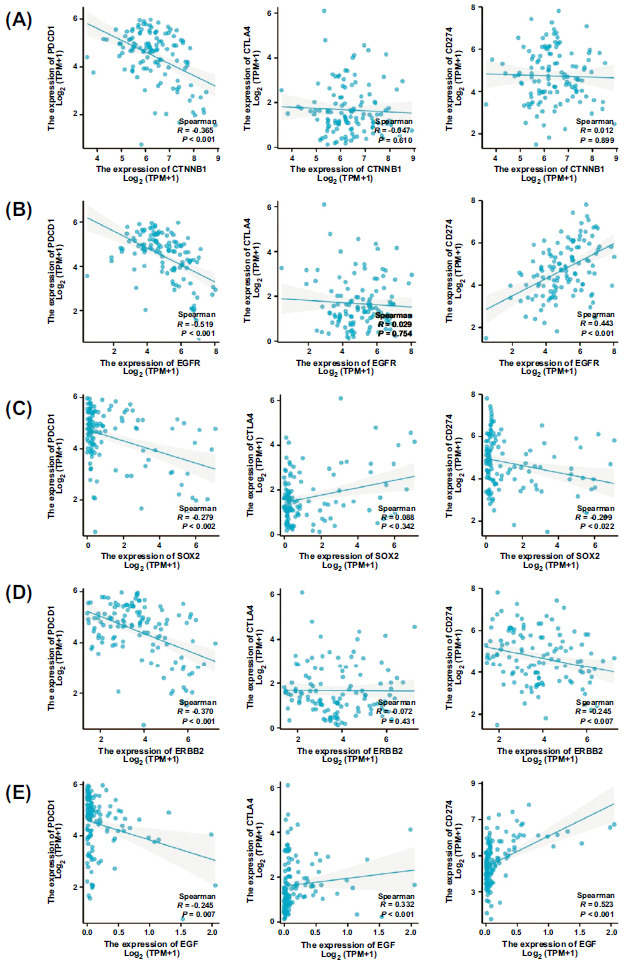
The relationship between 5 hub genes and the immune checkpoint-related molecules. (**A**) CTNNB1, (**B**) EGFR, (**C**) SOX_2_, (**D**) ERBB2, (**E**) EGF.

PDCD1, CTLA-4, and CD274 are common immune checkpoint-related molecules in antitumor therapy. Thus, our study explored the relationship between hub genes of TAMG and the immune checkpoint-related molecules. CTNNB1, EGFR, and ERBB2 expression levels all showed a significant negative correlation with PDCD1 expression levels (Fig. **6A**, **6B** and **6D**, R<-0.3). The expression level of EGF was positively correlated with the expression level of CTLA4 (Fig. **5E**, *R*>0.3). Both EGFR and EGF were positively correlated with CD274 expression (Fig. **6B** and **6E**, *R*<-0.3).

### 3.6 Hub Genes-miRNAs Interactions Prediction

NetworkAnalyst was used for the prediction of hub genes-miRNAs interaction. As shown in Fig. (**7**), hsa-mir-129-2-3p, hsa-mir-155-5p, hsa-mir-124-3p, hsa-mir-16-5p, and hsa-mir-21-5p may be the miRNAs that play regulatory roles upstream.

## 4 DISCUSSION

For patients with thymoma, the combination of MG affects the quality of life and survival time. In this study, we analyzed the DEGs between TAMG and NMG groups based on the TCGA database and identified 5 hub genes and their regulatory miRNAs. The correlation between the 5 hub genes and immune cell infiltration was also analyzed. Our findings could help partially clarify the pathophysiology of TAMG, which could be new potential targets for subsequent clinical immunotherapy.

CTNNB1 is a gene that encodes β-catenin, which is implicated in the canonical Wnt/β-catenin signaling pathway [21]. This is an evolutionarily conserved signaling pathway that is triggered in the presence of Wnt and leads to the activation of β-Catenin. β-Catenin controls tissue-specific gene expression in conjunction with several transcription factors [22]. Hepatocellular carcinoma (HCC) patients with CTNNB1 mutations frequently exhibit anti-PD1 therapeutic resistance. CTNNB1 mutations in HCC are associated with immunological exclusion [23]. T cells, B cells, M1-type macrophages, and dendritic cells (DCs) were among the immune cells that were dramatically downregulated in CTNNB1-MUT, whereas M2-type and macrophages were significantly upregulated and immunostimulant molecule expression was significantly downregulated [24]. Epidermal growth factor receptor (EGFR)/ERBB1, Erb-B2 Receptor Tyrosine Kinase 2 (ERBB2/HER2 in humans), Erb-B2 Receptor Tyrosine Kinase 3 (ERBB3), and Erb-B2 Receptor Tyrosine Kinase 4 (ERBB4) are the four members of the ERBB receptor family [25, 26]. In cells of the epithelial or mesenchymal lineage, EGFR signaling regulates cell proliferation, differentiation, survival, de-differentiation, and malignant transformation. It functions as a receptor for a variety of ligands, including amphiregulin (AREG) and epidermal growth factor (EGF). The catalytic Protein Tyrosine Kinase (PTK) activity of the ERBB1 is activated by one of its ligands, which also causes tyrosine phosphorylation of other related ERBB family receptors [27]. In lung cancer, EGFR-TKI resistance promotes immune escape in lung cancer *via* increased PD-L1 expression [28]. In gastric cancer and breast cancer, patients with high expression of ERBB2 have higher tumor malignancy and poorer prognosis [29]. In our study, EGF, EGFR, and ERBB2 were found to be the hub genes in TAMG, so ERBB receptor blockers may be a potential treatment for TAMG patients. The SOX family is involved in a number of early embryonic developmental processes, including the formation of the nervous system, the determination of sex, and the development of the lens [30]. As it is crucial for sustaining the stemness and self-renewal of embryonic stem cells (ESCs), SOX_2_ is the most significant of these SOX genes. The conversion of somatic cells into induced pluripotent stem cells (iPSC) is also largely dependent on SOX_2_ [31]. Yet SOX_2_ amplification and subsequent overexpression have also been shown in a variety of malignancies, including breast cancer, gastric cancer, and prostate cancer [32], raising the possibility that SOX_2_ is an oncogene. Small-Cell Lung Cancer (SCLC) develops in 50% of people with Lambert-Eaton Myasthenic Syndrome (LEMS), and SOX_2_ has been demonstrated to be an effective method for predicting SCLC in LEMS patients [33]. In our study, SOX_2_ was found to be among the 5 hub genes, so it can be concluded that SOX_2_ may also play a role in MG pathophysiology, warranting further studies in the future.

Th17 T cells promote inflammation by activating immune cells and facilitating their entry into organs, which, in turn, results in tissue-specific autoimmune inflammatory disorders. The pathogenesis of MG is significantly influenced by these cells. Multiple mechanisms are involved, including the release of interleukin-17 (IL-17) and other cytokines that subtly encourage the creation of immunoglobulin. According to several studies, MG patients have abnormal numbers of Th17 cells and IL-17, which are correlated with the severity of the disease and antibody titers [10, 34, 35]. By the ssGSEA method, we proved that CTNNB1, EGFR, SOX2, and ERBB2 expressions showed significant correlation with Th17 T cell in thymoma, and KEGG analysis suggested that differential genes were highly enriched in the IL-17 pathway. So, these four genes may have contributed to the development of MG by affecting TH17 cells. Natural killer cells (NK cells) can promote the differentiation of follicular helper T cells in myasthenia gravis [36], and myasthenia gravis patients with acute exacerbations have a reduced number of NK cells [37]. CTNNB1, EGFR, SOX_2_, and ERBB2 expressions revealed a significant correlation with NK cells in thymoma, and these four genes may play a role in regulating NK cells in TAMG.

Combined with the above findings, abnormal expression of 5 key genes may play an important role in TAMG patients. Aberrant expression of genes and immune cells in the tumor immune microenvironment of thymoma may be associated with the induction and maintenance of MG. In other words, a thymoma that develops into MG may depend on the heterogeneity of the thymoma tumor microenvironment. MiRNAs can influence gene expression by directly targeting mRNAs and inducing translational inhibition or instability. Growing evidence supports miRNAs as cancer diagnostic and therapeutic target genes [38, 39]. So, we predicted miRNAs that may regulate hub genes through an online database and obtained 5 possible regulatory miRNAs. These miRNAs could partially explain the heterogeneity of the thymoma tumor microenvironment.

There are some limitations in this study. First, this study was based on the TCGA database analysis, and the validation of clinical specimens was not performed. Second, the molecular mechanisms associated with 5 hub genes affecting immune cell infiltration were not investigated. These limitations will be explored and resolved in future studies.

## CONCLUSION

In this study, we identified 5 hub genes (CTNNB1, EGFR, SOX_2_, ERBB2, and EGF) and their 5 regulatory miRNAs in TAMG, and the hub genes were found to be correlated with multiple immune cell infiltrations and immune checkpoint-related markers. Our findings could help partially clarify the pathophysiology of TAMG, which could be new potential targets for subsequent clinical immunotherapy.

## Figures and Tables

**Fig. (4):**
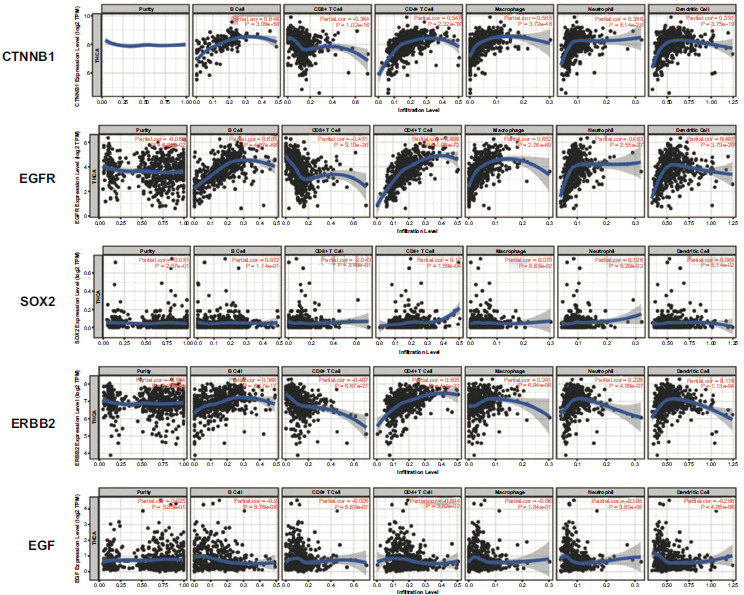
Correlation of five hub genes expression and immune cell infiltration in TIMER database.

**Fig. (7):**
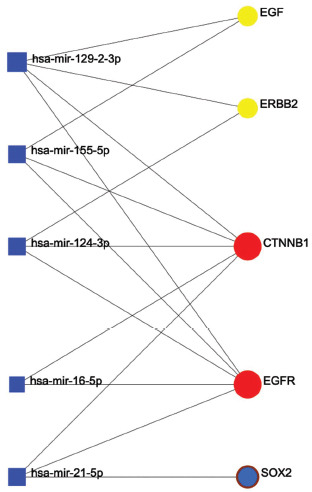
Prediction of hub genes-miRNAs interactions based on NetworkAnalyst.

## Data Availability

The data and supportive information are available within the article.

## LIST OF ABBREVIATIONS

TAMG: Thymoma-associated MG
NMG: Non-MG thymoma
TCGA: The Cancer Genome Atlas
KEGG: Kyoto Encyclopedia of Genes and Genomes
miRNAs: microRNAs
DEGs: Differentially Expressed Genes
