# Supplementary material for: Identification of Hub Genes and Analysis of their Regulatory miRNAs in Patients with Thymoma Associated Myasthenia Gravis Based on TCGA Database
Source: Microrna. 2024 Aug 26;14(1):49–58. doi: 10.2174/0122115366299210240823062457 (PMC12246741; doi:10.2174/0122115366299210240823062457)
Supplement: Supplementary file 1 — Supplementary material is available on the publisher's website along with the published article. [file MIRNA-14-1-49_SD1.pdf]

Supplementary Material

Identification of Hub Genes and Analysis of their Regulatory miRNAs in Patients with Thymoma Associated Myasthenia Gravis Based on TCGA Database

Wei Zhou[1,*], Jia Hu[2] and Jun Nie[1]

[1]*Department of Cardiothoracic Surgery, The First Affiliated Hospital of Wannan Medical College, Wuhu, China;* [2]*Department of Neurology, The First Affiliated Hospital of Wannan Medical College, Wuhu, China*

gene_name	log2FC	adjusted P value
AL772337.3	-6.10659	1.11E-09
RGS21	-5.86792	2.96E-09
CTSLP8	-5.86565	1.49E-11
OR9K1P	-5.85218	1.33E-08
AL353726.2	-5.8094	3.58E-11
LINC02428	-5.44729	1.37E-10
HOXC8	-5.24836	6.11E-13
SP8	-5.10373	4.23E-09
SOX14	-5.05498	8.24E-05
UGT2B28	-4.98894	0.014957
AL772337.1	-4.83326	4.8E-09
ZIC5	-4.73148	0.00095
TLX3	-4.68512	0.044603
LINC00408	-4.64468	1.09E-06
FAM41C	-4.62948	1.69E-07
AL772337.2	-4.61132	3.01E-06
AC090735.1	-4.56576	0.000157
SOX2	-4.52432	6.88E-14
AC099654.5	-4.4921	0.004996
ALOXE3P1	-4.47858	3.03E-05
MSLN	-4.4316	5.93E-10
HTR3C	-4.40352	0.000221
NEUROG2	-4.34118	0.00142
SOX21	-4.34008	2.16E-05
DBX2	-4.32955	1.25E-08
AL162574.2	-4.29723	3.09E-07
AL137001.2	-4.1842	5.84E-05

GS1-600G8.3	-4.17087	0.002723
AC004674.1	-4.16073	0.002201
NPY2R	-4.11916	2.34E-05
AC110813.1	-4.09966	5.59E-07
MAGEA1	-4.09836	0.009582
ZIC2	-4.09138	5.87E-06
IGF2BP3	-4.09117	4.93E-08
PROX1-AS1	-4.06322	3.67E-05
MYL1	-4.05967	0.003923
PLEKHG7	-4.05758	1.5E-06
FAM83A	-4.05635	1.53E-11
SOST	-4.04541	4.21E-05
PLA2G3	-4.04057	3.33E-06
MARCHF11	-4.03267	1.24E-05
AC104407.1	-4.03138	0.000272
MMP3	-4.01121	2.33E-10
TRIM51GP	-3.99313	0.000148
MSLNL	-3.98964	0.000699
AC092445.1	-3.98714	5.09E-07
FOXI1	-3.90224	6.56E-08
PROX1	-3.89631	1.48E-10
CCL20	-3.88752	1E-09
HEPACAM2	-3.86798	1.77E-09
NAALADL2-AS2	-3.86344	0.002091
AC010595.1	-3.85439	0.000208
ELF5	-3.77631	1.28E-10
LY6D	-3.75691	0.002851
SLCO1B1	-3.73291	0.000117
HOXD11	-3.72787	0.009023
AL138701.2	-3.72205	0.000209
CALHM3	-3.71602	2.79E-07
AC104794.4	-3.67605	5.05E-05
CCDC74BP1	-3.66406	1.03E-07
LINC01202	-3.64937	0.001848
HOXC13	-3.64226	2.15E-06
LINC01633	-3.6255	6.63E-07
ASCL3	-3.61739	9.44E-07
AC023310.4	-3.61306	1.22E-09
C11orf53	-3.6044	3.45E-08

CYP26A1	-3.58406	8.09E-07
HTR3E	-3.57473	1.48E-05
NDUFS5P2	-3.55338	0.005567
AC016044.1	-3.54026	0.007488
TRIM43B	-3.51943	1.82E-06
MMP13	-3.51056	1.08E-05
TRIM43	-3.49297	6.97E-08
NKX2-1	-3.48625	0.006007
GABRG2	-3.48193	0.000117
LINC02163	-3.47466	0.000204
FOXD1	-3.47206	3.45E-07
MAGEB6	-3.46188	0.01963
SFRP2	-3.44835	8.7E-08
GSX1	-3.43629	0.000291
CCNYL2	-3.43546	0.001009
AC087783.1	-3.41432	0.000259
AL121612.2	-3.41049	5.16E-06
PCSK2	-3.3994	1.84E-05
CFHR3	-3.39424	2.99E-11
SPATA31C1	-3.39372	6.05E-06
PYDC1	-3.37538	0.000853
FEZF2	-3.37462	0.002764
CALML5	-3.36235	2.04E-05
ALX1	-3.35996	0.000781
DLX6	-3.3458	1.32E-06
PAPPA2	-3.34403	2.12E-06
CHGA	-3.34137	1.82E-06
PPP1R1B	-3.3369	2.73E-10
SLC5A4	-3.32661	3.47E-07
IAPP	-3.32589	0.001545
DACH2	-3.32515	2.1E-07
LINC02228	-3.31706	4.22E-08
SLC5A7	-3.31347	0.002011
CFAP61-AS1	-3.31122	0.000127
AL133467.4	-3.30299	0.00079
HOXC10	-3.29789	0.000621
AL353726.1	-3.28581	0.000718
KIAA1549L	-3.27976	6.62E-10
OR5M11	-3.27535	0.000352

KRTAP4-1	-3.26772	0.003747
AL353572.2	-3.24816	4.46E-08
GNAT3	-3.2375	0.000993
ITPRID1	-3.22686	0.000306
AC091931.1	-3.22204	0.000646
LINC01456	-3.22117	9.15E-05
GTF2IP3	-3.20532	0.008403
LINC01203	-3.20472	6.28E-05
AC096656.1	-3.20034	0.000259
HOXB13	-3.18891	0.0088
PRAMEF20	-3.18128	0.001582
LINC01322	-3.17224	3.59E-06
LINC02575	-3.17033	2.79E-07
CHAT	-3.16917	6.42E-09
SPATA31E1	-3.16675	0.002197
USP24P1	-3.16557	0.00727
LINC00442	-3.16446	0.004023
ATXN3L	-3.1385	0.042624
TAS2R13	-3.1144	0.000129
CTCFL	-3.11074	2.44E-06
REG1A	-3.10433	0.000231
TRIM51FP	-3.10171	0.00121
LINC00524	-3.09559	0.017008
AC027287.1	-3.09368	0.00913
CYP24A1	-3.08413	1.66E-07
FOXD3-AS1	-3.08323	0.00058
PCAT7	-3.07413	5.62E-08
LINC01305	-3.06478	3.4E-05
AC096759.2	-3.06378	0.003591
AC011029.1	-3.05548	0.000349
DEFB4A	-3.04861	0.007433
PRAMEF12	-3.04676	0.000606
RNASEH1P1	-3.04349	0.000775
AC011370.1	-3.04023	1.82E-06
HOXC-AS2	-3.03867	0.001484
KLK6	-3.03506	2.39E-05
OTOP1	-3.03252	0.00104
AC018558.1	-3.01904	0.043745
LRP2	-3.01842	7.82E-10

LINC01254	-3.01266	0.027491
AC099499.1	-3.00824	0.001374
BNIP5	-3.00526	0.000553
NR2E1	-3.00412	1.35E-05
LINC02485	-3.00053	0.000821
GSC	-2.99403	2.75E-08
MAGEA4	-2.99031	0.034302
POU2F3	-2.9788	7.28E-08
ITGB6	-2.9764	4.9E-06
TRIM53CP	-2.9761	0.000216
AL161908.1	-2.97158	0.012449
TRIM53AP	-2.9711	0.006782
C10orf53	-2.96828	0.014083
GP2	-2.96602	0.048113
CLDN6	-2.96572	1.33E-08
B4GALNT2	-2.96542	0.005245
AC099792.1	-2.96369	0.000113
AL365256.1	-2.95033	4.14E-06
TBC1D3D	-2.94894	0.02204
KRT36	-2.94501	1.97E-11
AC008443.2	-2.94301	0.000137
ALOX15P2	-2.93938	0.011361
HOXC13-AS	-2.93311	0.000399
HMX1	-2.92694	0.003081
PITX1	-2.92673	2.02E-05
AC136759.1	-2.92619	8.68E-05
LINC02466	-2.92401	0.000636
ICAM5	-2.92174	5.69E-08
CPLX2	-2.91683	0.000242
AL772363.1	-2.90791	0.001201
DKK2	-2.90265	3.49E-08
AL353572.3	-2.90043	0.034746
ELF3	-2.89855	4.92E-09
NAP1L4P2	-2.89839	2.14E-05
MUC2	-2.89107	6.23E-05
HOXC6	-2.88582	2.56E-06
AC100823.1	-2.87716	0.000311
SLC18A3	-2.87711	0.018113
CACNG1	-2.86819	0.000115

AC009264.1	-2.86497	0.000348
LRRTM1	-2.86469	0.000339
TRIM43CP	-2.85868	6.45E-05
H3Y1	-2.85491	5.03E-06
RHCG	-2.85486	0.000656
LINC01518	-2.83047	7.04E-05
AC013640.1	-2.82995	0.02539
MYBPC1	-2.82822	2.21E-05
PDZRN4	-2.81817	0.000211
H3Y2	-2.80683	9.39E-05
KERA	-2.80654	0.000268
LINC02522	-2.80613	0.010885
LINC01297	-2.80081	0.001648
SMPD4P1	-2.79469	0.000188
AC114316.1	-2.79074	0.00189
PRTG	-2.78195	3.02E-07
OFCC1	-2.77972	0.000486
B3GNT3	-2.77845	2.09E-06
LINC02607	-2.77694	0.000305
OR1G1	-2.77529	1.04E-05
SLITRK3	-2.77072	0.003991
AC011840.1	-2.77063	0.03521
LINC02331	-2.7663	0.002313
NKX3-2	-2.7578	6.74E-05
TRIM49B	-2.75272	0.000351
DUX4L9	-2.7416	0.002723
AC073578.4	-2.73987	0.002934
KRT16P4	-2.73398	0.000184
CENPIP1	-2.73173	0.002511
ARHGAP36	-2.72827	2.37E-05
AC015522.1	-2.72783	0.004569
G2E3-AS1	-2.72374	0.000139
AC069540.2	-2.72212	4.75E-05
AL137804.1	-2.71393	0.000224
APOBEC1	-2.7133	0.025357
OXGR1	-2.70985	3.22E-07
AP001150.1	-2.70921	0.003934
SP9	-2.70619	0.025419
SPATA31C2	-2.704	2.07E-05

SOX21-AS1	-2.70066	0.000705
KCNJ18	-2.69925	0.002365
AC006372.4	-2.69918	0.000442
UGT8	-2.69704	2.22E-06
TFAP2B	-2.69157	0.000641
DEFB1	-2.68787	2.9E-06
LIN28B	-2.68635	0.045134
PLEKHS1	-2.68484	3.54E-06
NOL4	-2.68145	6.28E-05
FAM83E	-2.67865	4.01E-06
RN7SKP106	-2.67848	0.000784
LINC01981	-2.66972	0.000202
VGLL2	-2.66932	0.014835
AC025809.1	-2.65866	0.000803
TUBB8P11	-2.65474	0.000115
MAGEA11	-2.65465	0.01849
AC025881.1	-2.65246	0.004703
MUC4	-2.65073	3.76E-07
PHF2P2	-2.64978	0.011044
AC060834.1	-2.64255	0.002778
AC079298.3	-2.64116	0.000368
SLC6A10P	-2.6324	0.000644
HAPLN1	-2.6296	0.001194
GRM7	-2.62851	9.34E-06
TRIM49C	-2.62322	0.009558
LINC02005	-2.61703	0.000503
SFTPC	-2.61245	0.000446
EDIL3	-2.61235	1.82E-06
QRFPR	-2.60248	0.000118
LINC00460	-2.5993	0.000543
GNB3	-2.59459	1.73E-09
LBP	-2.59345	0.002239
PITX3	-2.59166	0.00823
EGFL6	-2.59101	4E-07
DUX4L26	-2.58846	2.19E-06
CD24	-2.58674	6.36E-07
AL034346.1	-2.58083	0.035759
AL691520.1	-2.57564	0.011086
IGLL4P	-2.57196	0.026076

AP001065.4	-2.569	0.001526
SOHLH1	-2.56003	0.031032
PRAMEF1	-2.55914	0.019363
LY6G6C	-2.55783	1.57E-06
OMD	-2.55694	1.73E-05
LINC01096	-2.55316	5.64E-05
HOXA11	-2.55102	0.006365
AC008969.2	-2.5467	0.000212
PRAMEF19	-2.54595	0.017104
TRPM5	-2.5451	7.22E-08
AC009271.2	-2.54446	0.000389
OR56A3	-2.53978	0.002058
VN2R19P	-2.53852	2.66E-05
GABRA3	-2.53304	1.18E-05
MYOG	-2.53028	0.010122
IGF2BP1	-2.52817	2.31E-05
MATN4	-2.52597	5.52E-05
AP003900.1	-2.51644	0.013524
FP326651.1	-2.5159	2.14E-05
HTR2C	-2.50951	0.010967
CRYM	-2.50906	1.35E-06
TMPRSS4	-2.50304	5.77E-06
NKX6-1	-2.50298	0.001776
ATP6V1G3	-2.50259	0.019462
WDR87	-2.48864	2.29E-05
RFX6	-2.48862	0.001932
PES1P2	-2.48316	0.009423
SMR3B	-2.48307	0.016608
SPAG6	-2.47771	7.72E-06
ADH1C	-2.47325	4.1E-05
AP000428.1	-2.46789	0.00287
CDX2	-2.46648	0.006471
SNX18P26	-2.46087	0.000391
AC018641.1	-2.46028	2.3E-05
LINC02475	-2.45482	0.008042
AC006206.2	-2.45457	0.000335
POPDC3	-2.45378	0.00221
PLAC1	-2.44876	0.001419
POTEF	-2.44468	8.85E-05

U3	-2.44347	0.001872
KCNJ13	-2.44215	0.000487
SLC30A8	-2.44145	0.000748
MYEOV	-2.44077	1.56E-07
ANKRD33	-2.43982	0.008387
AP005233.2	-2.43963	0.000517
AL160191.3	-2.43879	0.007433
TUFMP1	-2.43802	0.00565
AL606490.8	-2.43731	0.001998
TGM2	-2.43581	2.14E-07
TRPM2-AS	-2.43132	1.12E-07
HNF4A	-2.43096	0.003081
TMPRSS11A	-2.42835	0.003102
FAM240B	-2.42774	0.0096
CASC20	-2.4247	0.00725
SLC26A9	-2.42343	0.000505
VN1R54P	-2.41942	0.009336
AC107918.6	-2.41573	0.000662
IGF2-AS	-2.41081	0.001177
AC025580.1	-2.40689	0.01605
TAS1R3	-2.40484	2.22E-08
C1QTNF9	-2.40296	5.28E-06
LINC02797	-2.40277	0.002185
RFPL3	-2.39712	0.001042
HCAR2	-2.39437	1.54E-07
SLC9A3P2	-2.39276	0.002613
SAMMSON	-2.3823	7.72E-06
AZGP1	-2.3812	0.001076
CXCL13	-2.38009	0.00013
WDR72	-2.37987	0.00011
LINC00461	-2.37662	0.004695
MGAT3	-2.37324	1.68E-09
KCTD8	-2.37197	0.001245
AL390755.2	-2.37166	0.001027
PAX4	-2.37039	0.020342
AC011586.2	-2.37014	0.001255
SH2D6	-2.3698	1.08E-05
CLDN4	-2.36859	1.47E-05
FAM83A-AS1	-2.36629	0.002221

HCAR3	-2.36547	1.93E-05
STRA8	-2.36145	0.005105
AC115282.2	-2.35966	0.000217
CLDN3	-2.3583	1.76E-05
MED15P8	-2.3525	0.011466
AC237221.1	-2.35177	0.003284
CGA	-2.35063	0.009204
SFRP4	-2.34144	2.75E-05
RNU6-1178P	-2.34137	0.016508
SLC22A9	-2.3393	0.000119
LINC02444	-2.33527	0.003256
NUTM2F	-2.33251	0.003029
ZNF209P	-2.32678	0.030405
LINC02341	-2.32601	2.72E-07
STMND1	-2.3251	0.003158
PTGS2	-2.32287	5.51E-06
SRRM4	-2.3215	0.001873
TRBV20OR9-2	-2.32114	0.000205
NNAT	-2.32028	2.77E-07
TEX41	-2.31977	6.4E-07
SLCO1A2	-2.31868	0.000416
SSX3	-2.31736	0.021125
PURPL	-2.31599	2.63E-05
LINC02882	-2.31549	0.010062
AC018637.1	-2.31472	0.000352
ADAMTS20	-2.31407	0.002718
CST9LP2	-2.31174	0.000226
AL355516.1	-2.31133	0.044798
HYAL4	-2.3113	5.39E-05
HOXC9	-2.3076	0.000222
CCKBR	-2.30386	0.001006
KRT40	-2.30066	7.36E-06
AC012101.2	-2.29942	0.000623
ANKRD63	-2.29773	0.007639
AC007848.1	-2.29703	0.000294
AL049828.2	-2.29494	0.007433
AL035258.1	-2.29192	0.030071
AC012123.1	-2.29146	0.001193
FNDC1	-2.29103	3.76E-05

GJB7	-2.28786	8.74E-05
AC012494.1	-2.28783	0.000992
NTS	-2.28198	0.001418
OR7E145P	-2.27913	0.004832
FGF5	-2.27902	0.001422
AL031123.3	-2.27691	0.000334
PRAMEF2	-2.27677	0.047401
TMEM174	-2.27503	0.001993
AC126915.2	-2.27094	0.021482
SSX5	-2.26364	0.004838
RASAL1	-2.26326	1.26E-06
FOXP2	-2.26077	6.45E-05
HMGB1P36	-2.25972	0.008086
AP002383.3	-2.25623	8.89E-05
SNCB	-2.25545	0.017894
AL049695.1	-2.25265	0.003518
AC005993.1	-2.25085	0.000753
AC087393.1	-2.25062	0.009538
CYP4Z1	-2.24979	0.000261
AC009271.1	-2.24828	0.000544
AC034154.1	-2.24676	0.002604
EPYC	-2.24666	0.007039
GFI1B	-2.24393	9.89E-06
AC011700.1	-2.24284	0.038115
GPR158	-2.24221	0.003711
TWIST1	-2.2403	9.14E-06
LRAT	-2.24001	4.48E-06
CXCL1	-2.2396	0.000142
ERBB3	-2.23817	4.76E-05
AC073225.1	-2.23587	0.001149
PRB4	-2.23518	0.017142
TAF11L11	-2.23514	0.004097
CACNA1G	-2.23357	2.75E-06
KLHL38	-2.23179	0.001687
PTPRQ	-2.2306	0.002023
OTOGL	-2.22989	7.88E-05
AWAT1	-2.22768	0.001232
GPM6A	-2.22705	9.89E-06
AC093895.1	-2.22297	0.002825

SIGLEC15	-2.2229	3E-06
OR7D2	-2.22283	1.33E-05
AC022762.1	-2.21998	0.019693
CAPN13	-2.21764	0.010231
FAM9A	-2.2167	0.0147
AC087491.1	-2.21505	8.38E-05
CLDN8	-2.20955	0.007601
AL512358.1	-2.20928	0.036981
AL355834.2	-2.20795	9.62E-05
AC137894.1	-2.20793	0.000585
AC022140.1	-2.20329	0.003577
SLC9A2	-2.20307	5.87E-08
AL662791.2	-2.20156	0.014235
FOXA1	-2.19831	0.009507
OR7E12P	-2.1956	0.001489
WTAPP1	-2.19419	0.000184
PRAMEF8	-2.19365	0.017734
AC106738.1	-2.19328	0.008252
AL358613.2	-2.19121	0.023631
P2RX6P	-2.18935	0.015722
MUC16	-2.18838	0.000401
TSPEAR-AS1	-2.18829	6.3E-06
TSPEAR-AS2	-2.18804	1.6E-05
PINCR	-2.18789	0.000624
AL021877.2	-2.18171	0.017636
IQCM	-2.18128	0.000587
CLDN10	-2.18126	0.000167
PAX2	-2.18054	0.010704
ZAR1	-2.17905	0.004395
AL158211.4	-2.17896	1.79E-05
LL22NC03-63E9.3	-2.17672	0.000728
AC093893.1	-2.1696	0.002002
AL079305.1	-2.16886	0.000387
AC063952.4	-2.16583	0.001645
FRRS1	-2.1632	4.31E-10
NOS2	-2.16275	7.38E-06
PACRG-AS1	-2.16139	0.000118
OR7E85P	-2.16	0.002201
PI3	-2.15888	0.001531

PPP1R2C	-2.15797	0.024985
AP005242.4	-2.15771	0.005628
PRAMEF7	-2.1571	0.039556
GFRA3	-2.15433	5.72E-05
LINC00303	-2.15049	0.001859
AC099552.3	-2.15024	0.03845
AL033530.1	-2.14793	0.039581
AC092811.1	-2.14538	0.000216
AC078950.1	-2.14512	0.028121
SLC25A5P5	-2.14448	6.25E-07
LINC01967	-2.13958	0.00157
SERPINB7	-2.13898	0.001184
OGN	-2.13886	7.71E-06
AL390334.1	-2.13817	0.003827
TDRD12	-2.13486	2.27E-05
CYP2W1	-2.13214	0.000531
LINC01980	-2.13155	0.000498
CDH22	-2.13153	0.004731
IGLV11-55	-2.12971	0.027034
SLC34A2	-2.12776	0.00062
PIGR	-2.12596	0.000521
AC074389.2	-2.12418	0.001081
AL021920.2	-2.12153	0.019308
AC007846.1	-2.12087	0.036513
AL445072.1	-2.12012	0.001244
ALOXE3	-2.11746	8.72E-05
ZNF723	-2.11593	0.018164
TRIM49	-2.11584	0.033834
AC011504.1	-2.11467	0.004282
AC011287.2	-2.11168	0.034436
HOXC5	-2.10993	0.006875
SAR1AP1	-2.10895	0.019015
LINC00707	-2.10629	0.001439
LINC01587	-2.10474	0.004027
AC010275.1	-2.10439	0.001941
OR3A1	-2.10314	0.001941
C8orf34	-2.10254	1.36E-05
AP002954.2	-2.10151	0.004802
TRPC6	-2.09986	1.59E-06

OR2AF1P	-2.09842	0.00533
OR7E86P	-2.09753	0.018723
AC079753.1	-2.09642	0.007496
KRT16P5	-2.09612	0.01084
MUC5B	-2.09552	0.000307
SMIM5	-2.08612	2.48E-07
AC131180.1	-2.08581	0.032929
OTP	-2.08393	0.022626
AL157931.2	-2.0832	0.004916
SPINK1	-2.08157	0.004142
LINC01647	-2.07856	0.014169
AC124861.1	-2.0763	0.000689
AC091646.1	-2.07541	0.016482
AC106872.1	-2.07081	0.011793
FBP2	-2.07001	0.000272
AL606500.1	-2.06897	0.001499
PITX2	-2.06883	0.000189
AL451142.1	-2.06851	1.1E-05
ALOX12B	-2.06849	5.83E-05
CYB5R2	-2.06724	9.69E-08
ADAMTS19	-2.06719	0.001953
IGHV1-67	-2.06587	0.003699
SYT12	-2.06408	2.23E-06
CREB3L1	-2.06332	2.26E-05
AC027584.1	-2.06069	0.005257
AC109635.3	-2.05929	0.029633
ANO1	-2.05894	9.15E-06
LIX1	-2.05469	0.008455
GABRR1	-2.05303	1.7E-05
AC097462.3	-2.05064	0.002624
AC100782.1	-2.04925	0.003029
CHI3L1	-2.04658	4.66E-05
PLA2G2A	-2.04564	0.019383
ITGB8	-2.04198	8.41E-05
ASNSP1	-2.04179	0.017142
LRRN4CL	-2.04046	2.26E-05
MGAT3-AS1	-2.03856	0.01007
AC026408.1	-2.03493	0.001317
TUBB2B	-2.03424	7.42E-06

LINC01901	-2.03344	0.008833
TENM2	-2.03247	0.000121
OTX1	-2.03104	0.006779
AC106875.1	-2.03002	0.044397
PLCE1-AS1	-2.02818	0.003284
AC009227.1	-2.02695	0.006453
AC108673.1	-2.02693	0.010517
FAM90A27P	-2.02674	0.034704
TACSTD2	-2.02559	1.99E-05
LINC02303	-2.02427	0.025433
AC108676.1	-2.02282	0.000451
CACNA1B	-2.02056	0.000196
TRIM48	-2.01252	0.01711
AC007128.1	-2.0119	0.005353
AC008464.1	-2.00848	0.046759
SVEP1	-2.00766	4.09E-06
NOTUM	-2.00705	8.58E-05
AC092666.1	-2.00703	0.009161
CHRM1	-2.0049	0.040793
ZNF729	-2.00419	0.046622
MIR4482	-2.00352	0.000262
SLC35G3	2.006115	6.3E-08
AP003484.1	2.007186	0.003326
AL022337.1	2.009396	0.007433
FAM95C	2.010001	5.81E-10
LINC01767	2.012013	3.7E-05
AC006013.1	2.012226	0.004589
RPL35AP20	2.012424	0.000148
AC093330.1	2.013347	0.000216
ALDH1L1-AS2	2.015044	6.52E-05
AL590556.2	2.016096	0.000258
LINC00668	2.020595	0.001207
AL391361.2	2.021507	0.001303
AC069281.1	2.022535	4.66E-05
GRHL3-AS1	2.023252	0.004454
ALDH4A1	2.025224	7.09E-10
HSD3BP2	2.025807	0.017273
AL357873.1	2.027336	8.3E-09
SFTPB	2.027458	0.000399

HMGB1P29	2.027923	0.03396
DSG4	2.029591	0.009463
AC015720.1	2.031143	0.042606
MTOR-AS1	2.033564	0.000385
SALL3	2.03547	0.035248
STRC	2.036485	1.96E-06
RNF148	2.038603	1.99E-05
RPS3AP54	2.038683	0.00775
CD300LD	2.042866	0.000637
AC064805.1	2.046573	3.5E-06
KRT6B	2.046997	0.004437
AC084024.3	2.047076	0.004215
SLC34A1	2.047518	0.00028
ANGPT4	2.049187	6.86E-07
C4orf50	2.051728	0.004608
AL035425.3	2.06048	0.021622
PPARGC1A	2.060771	1.81E-08
AL353600.2	2.060945	0.010108
ATP10B	2.061476	0.004148
AL135818.3	2.065344	3.39E-08
AKR1B10P1	2.065348	0.015014
MYH13	2.071128	6.13E-05
LINC00640	2.073024	1.6E-06
GAS2L1P1	2.073953	0.000102
LINC02074	2.074667	0.001903
DSG1-AS1	2.075974	0.0003
CER1	2.076859	0.032072
AL031429.1	2.081417	0.007926
FAM183BP	2.085358	9.38E-06
FEM1AP2	2.086859	0.004063
LINC00964	2.091584	0.000297
TCERG1L	2.091874	2.6E-06
AC012640.3	2.095192	2.27E-05
GDAP1L1	2.096049	1.5E-06
AL355482.1	2.096617	1.91E-05
TRPC5	2.100573	0.004829
AC015660.5	2.103747	0.004191
AL160411.1	2.105122	0.035546
AP005902.2	2.105554	0.005209

TEX13C	2.106116	0.034257
MYHAS	2.109734	0.00391
FBXW12	2.109938	1.03E-07
S100A8	2.112113	5.92E-06
POM121L2	2.113473	0.001903
PCDHB17P	2.114052	0.002746
FGF10	2.11443	0.000339
FAM166A	2.114464	1.03E-10
KRT32	2.114824	3.97E-06
CASC16	2.119243	0.019591
RPL6P5	2.122675	0.011284
LINC01755	2.124109	0.005097
RBFOX1	2.124134	1.15E-05
USP26	2.12417	8.72E-05
PTCHD1	2.124315	0.002808
TRIM7	2.124982	1.48E-10
AC123777.1	2.127259	0.001973
EFCAB6-AS1	2.127693	3.29E-07
PRSS38	2.129959	0.000138
LINC01309	2.130231	0.021833
LINC02635	2.133886	0.004762
NPC1L1	2.136566	1.32E-09
AL033381.2	2.13721	0.001183
AL713852.1	2.137827	1.87E-05
ALDH1A1	2.140259	7.99E-07
LINC01267	2.14069	0.009001
MED15P3	2.141986	0.029676
LINC02800	2.142123	0.002163
GSG1L	2.144905	3.21E-05
AL356489.3	2.149039	3.85E-06
ANKRD18CP	2.1491	1.83E-08
AC007741.1	2.149419	0.014458
SPOCK1	2.153047	0.001938
PXDNL	2.155155	1.05E-05
AL049833.2	2.157879	0.003038
AC112219.2	2.158899	0.001957
LINC00332	2.159078	0.000495
UGT2B7	2.163261	0.035549
ACP7	2.165183	0.000569

AC084262.2	2.167085	0.000909
AC005042.2	2.168373	0.001844
AC130686.1	2.168639	3.44E-06
RPL13AP17	2.169841	0.023834
OR2M4	2.17029	0.00014
AC009236.1	2.170521	3.68E-06
AC138356.3	2.171614	0.00277
AC093833.1	2.175258	0.000365
AC120498.8	2.176848	0.02434
AL079301.1	2.180088	0.026873
FAM3D-AS1	2.181733	6.77E-09
SEZ6L	2.185196	0.000109
CNKSR2	2.18615	1.5E-13
APOH	2.193199	0.007639
AL160410.1	2.193435	0.003551
AC005343.7	2.196338	0.004062
CARTPT	2.198846	0.003475
LINC02555	2.202556	0.000338
AC009041.1	2.203061	1.22E-05
AC010280.3	2.206048	0.017985
AC015660.2	2.206317	1.61E-07
AF106564.1	2.207528	0.000385
AC087283.1	2.208584	0.009457
AC092818.1	2.209686	0.001835
RNA5SP342	2.216496	0.000544
PEX5L-AS2	2.221494	1.1E-05
CA3-AS1	2.223095	3.64E-11
SLC22A4	2.223658	2.06E-12
IGFBPL1	2.227339	3.95E-06
AC022034.3	2.228096	0.001579
AL117372.1	2.228467	0.001079
LINC02717	2.229755	0.002023
BX005266.3	2.231746	0.027518
AL390726.5	2.233706	1.02E-05
DNAH9	2.234185	2.76E-08
HIKESHIP1	2.234921	0.010804
ENOX1	2.235482	2.45E-11
ACOT6	2.237377	6.25E-08
KCNC1	2.243145	7.46E-05

LASTR	2.243223	7.04E-06
FATE1	2.245006	8.43E-06
AL356277.2	2.246167	0.048033
SLC26A5	2.247082	0.000123
CDC20P1	2.24786	1.26E-10
KLHDC8A	2.248641	8.15E-08
PKD1L2	2.250067	6.35E-09
AL391844.1	2.253718	1.09E-06
AL008718.2	2.254351	1.31E-10
AC022916.3	2.254431	1.1E-05
DGCR5	2.261457	6.84E-08
RPS4Y2	2.266325	0.002214
RAX2	2.27055	0.003102
F11	2.273717	0.003704
PRLH	2.277119	0.032208
AC090192.2	2.277339	0.00013
LINC02139	2.277984	3.08E-08
OR10H1	2.281205	0.000124
GRIA3	2.285813	0.001159
AC097515.1	2.285974	0.000148
AC140479.2	2.28703	0.000186
POU3F1	2.288914	9.57E-09
AC025284.1	2.290115	2.04E-07
AL353681.1	2.294258	0.007393
AC112206.2	2.300711	3.01E-06
AC092745.1	2.305188	0.006669
AC096741.1	2.306195	0.000779
C14orf180	2.30678	0.030608
AL162725.2	2.308044	0.011429
AC010608.1	2.308499	1.22E-05
AL512658.2	2.30994	0.005039
AC005532.1	2.310332	8.88E-12
DSG1	2.311918	1.47E-06
AC107892.1	2.316388	0.009319
LINC02687	2.316776	0.00185
ZEB2P1	2.321036	0.000178
CLCA2	2.321136	7.76E-05
RPL35AP6	2.330761	0.010828
AL009178.1	2.34257	0.023418

LRRN3	2.345809	3.33E-08
BCHE	2.346513	0.000124
AC011995.1	2.349803	0.016098
RBFOX3	2.351554	3.63E-07
AL355538.1	2.356182	0.001292
ENPP3	2.359864	4.07E-06
PRDM12	2.363155	8.35E-06
RN7SL233P	2.364158	0.000153
AC010420.1	2.364491	0.002503
LINC02471	2.364928	0.000452
AC008571.2	2.371826	0.035758
MAP2	2.372741	1.93E-12
AC099509.2	2.37379	0.016937
LINC01649	2.377685	1.76E-06
PTN	2.377713	9.63E-07
AC107373.1	2.377733	0.002257
AL133516.1	2.378479	0.021482
SYNDIG1L	2.380435	4.91E-09
AL162713.2	2.383232	4.91E-09
LINC02242	2.383424	2.24E-12
MTUS2	2.386001	1.16E-07
AC006427.2	2.386282	5.34E-06
ASS1P9	2.393378	8.65E-05
ADAMTS18	2.393653	1.7E-09
OR13J1	2.402497	1.66E-05
AC092637.1	2.402534	0.000682
AC011995.2	2.408236	2.13E-07
EGF	2.408353	2.36E-06
LINC02501	2.409296	0.001602
LINC01170	2.409842	0.011957
AC010547.2	2.412947	0.001345
TCEA1P3	2.415437	0.000155
AC116312.1	2.415959	1.19E-06
TF	2.416776	8.51E-10
FAM47DP	2.418013	0.000175
LINC02200	2.420034	0.005073
LINC02853	2.420277	3.7E-08
PHF24	2.421802	1.28E-07
FAM135B	2.425705	7.41E-06

AC078785.3	2.42592	3.68E-05
LINC01341	2.429702	1.7E-09
VN1R51P	2.42976	1.69E-07
PPIAP84	2.433493	7.99E-06
NOS1	2.434841	0.000124
MTCO3P12	2.437394	5.23E-11
SFTA1P	2.445887	0.000311
CYP4F3	2.447348	4.27E-05
PDE1A	2.450295	4.45E-07
AL139147.1	2.451482	0.006024
KC877982.1	2.453962	0.012481
C2orf66	2.456106	1.67E-12
BX119927.1	2.462754	0.003241
KRT6C	2.4632	0.001776
TMEM163	2.466333	1.62E-09
CYP2C9	2.470637	0.00318
LINC00519	2.473994	0.000179
AC112693.1	2.474085	1.47E-05
FIBCD1	2.478837	0.000605
KBTBD12	2.484669	1.5E-13
LINC01250	2.485763	0.000136
AP002800.1	2.497215	1.81E-08
LINC02159	2.498268	5.35E-05
ARMC8P1	2.498571	0.000569
AL359399.1	2.503619	6.32E-05
SULT2B1	2.503865	1.29E-08
AL162717.1	2.504895	6.57E-05
AL596087.2	2.505123	0.002893
LINC01672	2.510277	0.006207
MIR6510	2.517176	0.003862
AL031291.1	2.53464	0.001451
KRT15	2.538805	6.18E-06
AC008443.3	2.5481	2.45E-11
LINC01166	2.549043	0.005479
AC005042.1	2.551509	0.000118
LINC01750	2.551597	9.54E-06
DPYS	2.551811	5.5E-05
SORCS3	2.555709	0.000417
AL021328.1	2.556005	0.003725

AL357137.1	2.558687	2.41E-05
AL157996.2	2.563776	0.000344
LINC02209	2.567487	0.034582
GABRE	2.567656	4.04E-09
AC005725.1	2.572116	1.44E-05
THRSP	2.574366	1.05E-05
AC026469.1	2.574378	6.49E-07
LINC02473	2.57624	0.004604
IRS4	2.576635	0.006606
LINC01500	2.580999	9.56E-06
AC091979.1	2.58453	3.09E-07
LINC02518	2.587814	7.26E-05
AC131902.1	2.588264	0.000749
LINC01179	2.592004	0.007765
YWHABP1	2.594529	1.52E-08
LINC01823	2.595295	2.03E-08
AL357127.1	2.59752	0.004951
AC018716.2	2.598224	0.007801
AC010998.1	2.601307	0.000199
AC122136.1	2.602652	0.008525
AL136369.2	2.604142	0.001965
CCNYL5	2.604892	0.001945
OR2T4	2.617455	0.036155
AC084212.1	2.618527	0.000175
TRPC5OS	2.621555	3.49E-08
AC010307.4	2.623438	3.69E-10
AC018563.1	2.625395	2.21E-05
AC139426.1	2.629936	0.009793
LINC00844	2.632428	9.6E-05
AC084277.1	2.637378	7.11E-05
AL158152.1	2.637525	0.00021
AL589645.1	2.637559	2.44E-06
CLDN19	2.637641	1.89E-06
IL13RA2	2.640982	5.92E-09
AL590560.5	2.643693	0.00244
PCSK1	2.656807	6.7E-08
VEGFD	2.658029	1.29E-08
PCP4L1	2.658555	2.44E-06
SEC14L6	2.65948	9.1E-07

AC025470.2	2.660021	0.001475
LINC00589	2.668482	0.000803
SLC6A1-AS1	2.669195	5.81E-05
AC062015.1	2.669801	1.57E-06
AC093607.1	2.675115	0.001897
SPHKAP	2.679056	0.000108
FAM71F1	2.681299	1.06E-07
CAMK2B	2.68224	9.62E-08
MEGF11	2.68933	8.03E-07
NPBWR1	2.690374	1.01E-06
NRG1-IT1	2.692341	0.027106
SFTPA1	2.704441	0.001316
AL139393.1	2.704877	1.51E-07
CSNK1A1P1	2.705635	5.65E-08
Z82186.1	2.707053	0.003509
AL136114.1	2.708815	0.006015
FYB2	2.710835	6.49E-07
ADGRD2	2.714905	0.000631
AC135731.1	2.719324	0.000966
OLIG2	2.723434	0.006712
ENOX1-AS1	2.723436	0.000226
IL31RA	2.725681	1.37E-05
SHISA6	2.727629	1.68E-09
RYR3	2.734947	1.48E-13
AC009988.1	2.734984	0.000137
BCL9P1	2.739882	3.67E-12
CXorf49B	2.740221	0.000185
AC090572.3	2.747436	0.004838
AC093606.1	2.747509	0.001425
AC027288.3	2.75172	4E-07
RGS4	2.751988	2.83E-10
LINC02512	2.753712	0.030766
RTL4	2.756276	0.007231
AL512785.1	2.759617	0.002723
GLDCP1	2.761406	0.040806
ASIC2	2.776229	0.001151
PLXNB3	2.778892	2.36E-10
GBP6	2.78534	5.15E-06
GUCY2F	2.792628	0.000213

GABRA5	2.813243	7.43E-06
AC010329.1	2.813651	0.000261
GRM7-AS1	2.819476	0.008483
LINC00604	2.83947	0.000569
MTCO3P41	2.841807	0.004062
TCTEX1D1	2.845632	4.85E-11
AL031123.5	2.84625	3.67E-12
AC078816.1	2.84628	0.000723
OR7E130P	2.849146	3E-06
LINCR-0003	2.850813	0.004771
AC023421.1	2.86843	2.29E-13
AC009236.2	2.88491	3.23E-07
SGIP1	2.887387	3.4E-13
HGD	2.890216	7.32E-09
AC080079.1	2.911912	0.000659
LINC02629	2.916043	3.78E-06
AP002755.1	2.924526	1.12E-07
AL160262.1	2.931817	4.66E-05
OLFM3	2.937531	0.000113
FABP6-AS1	2.938066	0.000307
TRIM67	2.938308	2.52E-09
LINC02741	2.941281	0.016123
AL096709.1	2.944092	9.94E-05
AC079804.3	2.958089	4.25E-06
KIF28P	2.970466	6.88E-14
PAFAH1B2P2	2.982944	8.02E-05
FSHB	2.989239	0.006112
SMPX	2.997596	5.66E-08
AC137936.2	2.99781	2.06E-14
LINC01753	3.007941	0.001003
AP003119.1	3.012583	8.02E-08
BBOX1	3.021053	6.88E-14
ABCC12	3.040481	1.31E-05
SLITRK6	3.049631	4.16E-06
AC079467.1	3.060827	0.001434
OR10AC1	3.063871	1.38E-07
AC018467.1	3.066288	1.73E-09
AL807742.1	3.071863	7.3E-09
AC005482.1	3.073967	0.004615

VN1R48P	3.075572	4.53E-09
TRAJ24	3.091672	7.66E-12
Y_RNA	3.092706	8.61E-06
LINC02153	3.098763	0.00138
LINC02641	3.117554	5.71E-16
S100A9	3.129861	2.15E-10
NEFM	3.130667	1.52E-05
GLRA4	3.134682	1.57E-06
LINC00930	3.147064	3.11E-14
VWC2	3.152425	2.51E-08
AL162713.1	3.16112	3.1E-08
PHEX-AS1	3.182599	1.02E-05
AC084759.3	3.21174	8.51E-05
AC008163.1	3.236124	1.82E-05
AC018541.1	3.244258	0.000102
OR2A4	3.248219	4.5E-06
ITLN1	3.27672	2.86E-07
LIPF	3.308921	0.000196
CAPN6	3.310759	5.8E-05
YAP1P2	3.315369	0.000349
PLEKHD1	3.327516	1.55E-11
RBMXL3	3.338399	0.005674
AL035425.4	3.345956	0.00727
AL357127.2	3.346917	1.72E-07
LINC02786	3.389987	0.000467
AC093775.1	3.396046	2.98E-07
AC131254.1	3.408118	4.46E-07
INSYN2B	3.41657	3.79E-10
LINC02247	3.425379	1.96E-06
FSHR	3.448137	0.000604
AL354863.1	3.448241	0.00687
GLULP4	3.451417	9.73E-10
ANKRD30B	3.45153	0.000182
AC080079.2	3.459672	6.36E-07
AC009975.2	3.465088	0.000102
LINC00879	3.471492	0.009715
LRTM2	3.486482	2.03E-05
EPHA5	3.502607	1.89E-06
FAM189A1	3.5338	1.95E-09

CEND1	3.580617	1.5E-13
NOL8P1	3.591984	1.13E-06
AL356515.1	3.626741	3.38E-05
AC104137.1	3.659695	0.001151
CSMD3	3.695919	7.16E-07
LINC00940	3.718302	4.17E-10
LHFPL4	3.719922	4.46E-06
AC055874.1	3.73569	4.91E-09
AC010086.3	3.747276	0.001045
GLDC	3.79175	8.17E-22
LINC00681	3.927498	1.17E-05
NGB	4.551493	7.2E-14
AL928596.1	4.560541	2.49E-07
AL163195.2	4.686819	5.51E-06
